# Respiring to infect: Emerging links between mitochondria, the electron transport chain, and fungal pathogenesis

**DOI:** 10.1371/journal.ppat.1009661

**Published:** 2021-07-08

**Authors:** Braydon Black, Christopher Lee, Linda C. Horianopoulos, Won Hee Jung, James W. Kronstad

**Affiliations:** 1 Michael Smith Laboratories, Department of Microbiology & Immunology, University of British Columbia, Vancouver, Canada; 2 Department of Systems Biotechnology, Chung-Ang University, Anseong, Republic of Korea; University of Maryland, Baltimore, UNITED STATES

## Introduction

**Fig 1:**
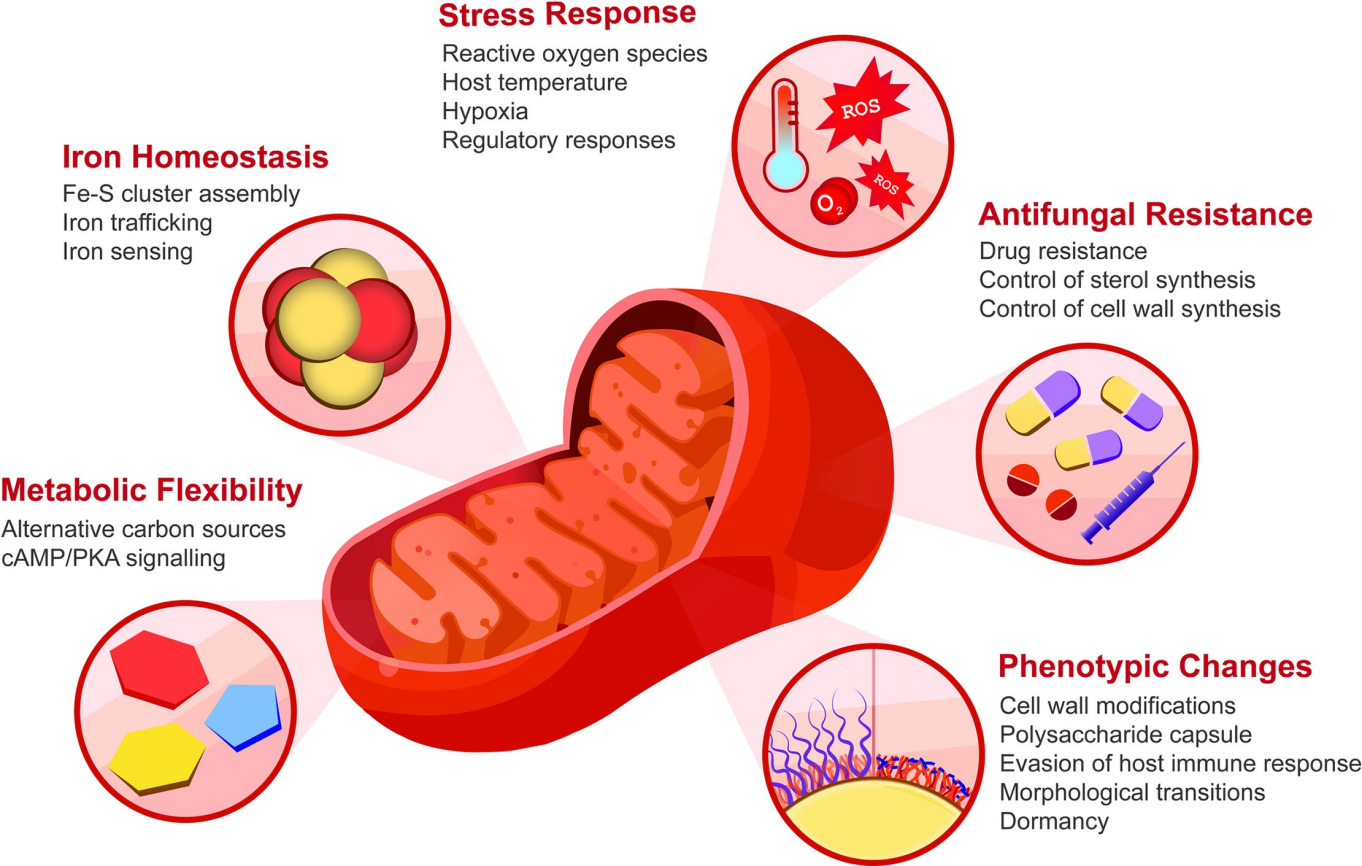
Mitochondrial-associated activities that impact fungal pathogenesis. Mitochondria play key roles in stress responses and metabolic activities (e.g., iron homeostasis and use of carbon sources) relevant to fungal proliferation in mammalian hosts. Recent studies emphasize the prominent participation of the ETC in virulence, and the targets of inhibitors of specific complexes are shown in Fig 2. The phenotypic outcomes of perturbed mitochondrial function include cell surface changes that directly influence evasion of the host immune response. Critically important activities related to drug susceptibility and resistance are the subject of other recent reviews [2–9]. cAMP, cyclic adenosine monophosphate; ETC, electron transport chain; PKA, protein kinase A; ROS, reactive oxygen species.

**Fig 2:**
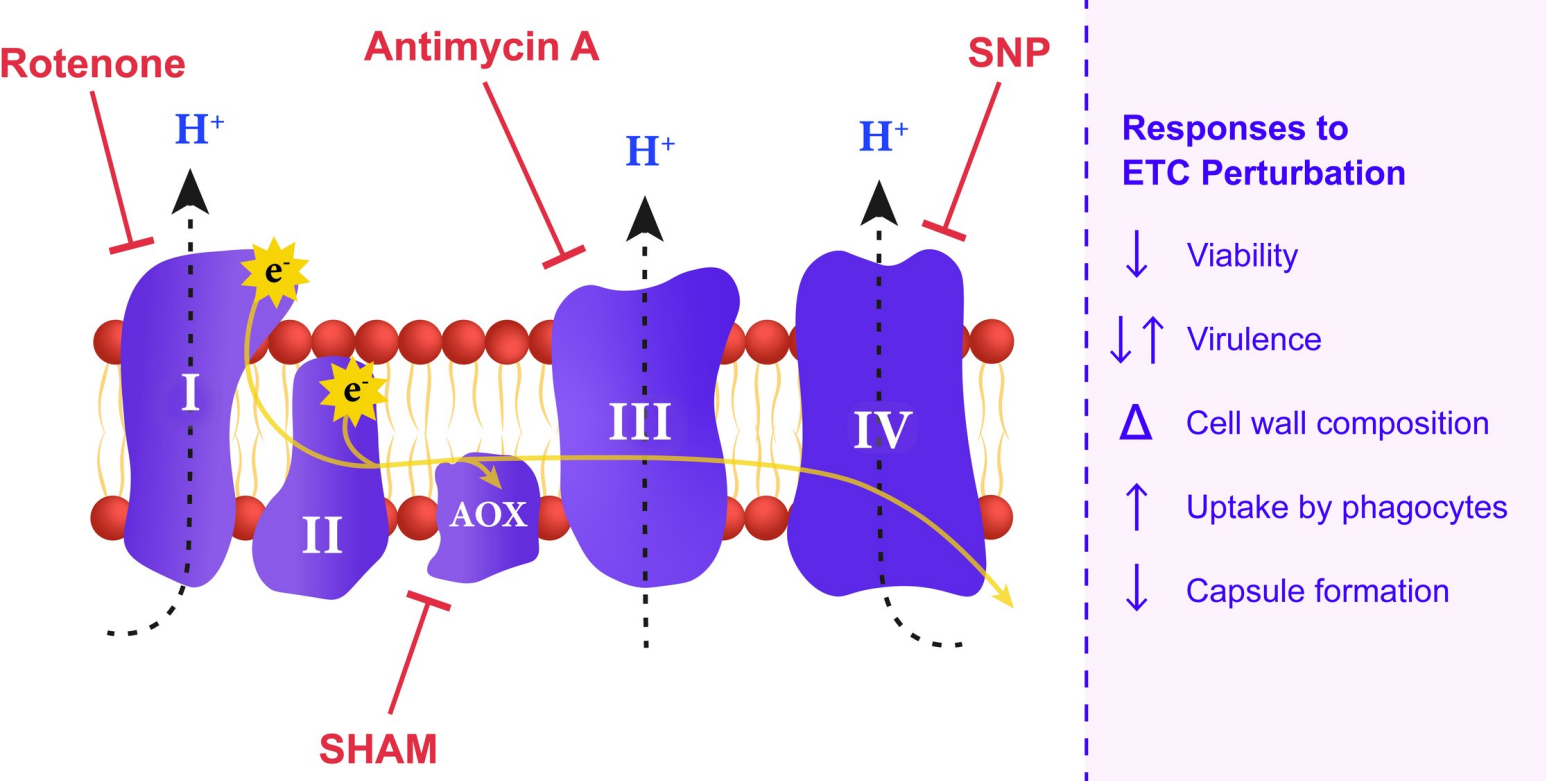
Inhibitors of ETC components provoke a variety of responses in fungal pathogens. A schematic illustrating the respiratory chain protein complexes in the inner mitochondrial membrane is shown along with the inhibitors that target specific complexes, as highlighted in the text. Selected examples of the key phenotypic responses to the inhibitors are indicated on the right. Note that capsule formation is specific to *C*. *neoformans*. ETC, electron transport chain; SHAM, salicylhydroxamic acid; SNP, sodium nitroprusside.

Fungal pathogens threaten human health both directly as infectious agents and indirectly by limiting crop production, and new approaches are desperately needed to combat fungal diseases [1]. There is a growing appreciation that mitochondrial functions contribute to the ability of fungal pathogens to cause disease and may be promising targets for new therapeutic approaches [2,3]. A number of excellent reviews provide insights into the roles of mitochondria in fungal pathogens; readers are directed to these reviews for information on connections between mitochondria and virulence, antifungal drug resistance and susceptibility, and cell wall synthesis [4–8]. Our intention in this review is to focus on recent studies that highlight mitochondrial connections to virulence, metal homeostasis, the response to stress, and metabolic adaptation, as summarized in Fig 1, for a selected set of fungi that are major agents of human disease: *Aspergillus fumigatus*, *Candida albicans*, and *Cryptococcus neoformans* [9]. *A*. *fumigatus* is the causative agent of noninvasive pulmonary infections (aspergillomas and chronic aspergillosis), allergic bronchopulmonary aspergillosis, and invasive pulmonary aspergillosis. *C*. *albicans* is a commensal in the human gut but can cause invasive candidiasis of the blood stream and internal organs, as well as diseases involving mucosal surfaces. Infections with *C*. *neoformans* generally begin in lung tissue, but the fungus has a propensity to disseminate to the brain to cause meningoencephalitis, a disease that is highly prevalent and often fatal in the HIV/AIDS population. These fungi have aerobic lifestyles dependent on mitochondria, and the involvement of the electron transport chain (ETC) emerges as a common theme in their ability to cause disease (Fig 2) [5,10,11].

### The electron transport chain is connected to fungal pathogenesis

Mitochondrial function is important for fungi to cause disease, particularly in the context of adaptation to challenges in the mammalian environment (e.g., nutrient limitation, the response to stress, and the immune response). However, the complexities of determining the specific roles of mitochondria are clearly illustrated by a recent study showing that inhibition of respiration actually increased the ability of *C*. *albicans* to cause disease in a mouse model of systemic infection [12]. This study employed the nitric oxide donor sodium nitroprusside (SNP) to inhibit classical respiration at complex IV in combination with inhibition of the alternative oxidase in the alternative pathway with salicylhydroxamic acid (SHAM) (Fig 2). The goal was to investigate whether these agents had antifungal efficacy by targeting respiration, given their low toxicity in other therapeutic applications. Together, SNP and SHAM impaired cell viability and triggered sensitivity to the agents calcofluor white and congo red that challenge cell wall integrity. An impact on the expression of cell wall functions was established by transcriptome analysis of treated cells, and an examination of structural and composition changes for the wall indicated increased exposure of chitin and β-glucan. Fungal cells pretreated with the inhibitors were taken up at a higher rate by the murine J774.1 macrophage-like cell line and by macrophages in a zebrafish model. Similarly, increased phagocytosis was observed by Cui and colleagues upon treatment with the complex III inhibitor antimycin A but not with inhibitors of other complexes in the ETC [13].

Increased virulence in a mouse model of cryptococcosis is also observed for a mutant of *C*. *neoformans* with an insertion in the promoter region of the gene for mitochondrial NADH dehydrogenase, a component of complex I of the ETC [14] (Fig 2). In this case, the mutant was obtained by serial passage in the wax moth *Galleria mellonella*, and subsequent characterization revealed an increase in the transcript for the gene as well as increased ATP production and metabolic rate. Interestingly, the mutant also had enhanced production of 2 key virulence factors for *C*. *neoformans*, the polysaccharide capsule and cell wall melanin. Other studies have linked mitochondria function to capsule formation including a demonstration that the ETC inhibitors rotenone, SHAM, and antimycin A reduced capsule size [15,16] (Fig 2). Overall, these studies in *C*. *albicans* and *C*. *neoformans* illustrate the complex impact of mitochondrial functions on the ability of these fungi to cause disease.

### Mitochondria play a central role in iron homeostasis for fungal pathogens

Mitochondria are key organelles for iron metabolism because the metal is a cofactor in proteins that contain iron–sulfur (Fe–S) clusters, heme, or iron ions, and many of these proteins function in respiration [17] (Fig 1). Iron sensing and acquisition are crucial for the proliferation of fungal and other microbial pathogens, and competition with the host for iron is an important aspect of disease [6,18–20]. In a clear demonstration of connections between iron and virulence, iron limitation was found to alter the cell wall of *C*. *albicans*, resulting in masking of β-glucans and evasion of the immune system (Fig 1) [21,22]. The response to iron limitation was dependent on the iron permease, Ftr1, cyclic adenosine monophosphate (cAMP)-dependent protein kinase A (PKA), and the transcription factor Sef1 that activates the expression of iron uptake functions and is required for candidiasis [21]. In addition, loss of the mitochondrial fusion protein Fzo1 results in elevated mitochondrial iron levels, constitutive expression of the Sef1-regulated iron regulon, a defect in Fe–S cluster synthesis, and retention of Sef1 in the nucleus [23]. These observations are consistent with earlier studies in other fungi (including *A*. *fumigatus*) demonstrating that iron sensing involves a regulatory signal released during mitochondrial Fe–S cluster assembly [19,24]. Interestingly, cytosolic Fe–S cluster assembly (CIA), a pathway involved in maturation of cytosolic and nuclear Fe–S proteins, is dispensable for iron sensing in both *A*. *fumigatus* and *Saccharomyces cerevisiae* [24]. Together, these data suggest a vital role for mitochondrial Fe–S cluster assembly in iron sensing and regulation by fungal pathogens.

Mitochondrial transporters and functions for intracellular trafficking of iron and heme are also important for fungal pathogenesis [25–28]. For example, the use of a genetically encoded heme sensor demonstrated that inhibition of mitochondrial respiration reduced the labile heme pool in the cytosol of *C*. *neoformans* [29]. Furthermore, iron acquisition, cell wall integrity, mitochondrial function, and virulence in *C*. *neoformans* are dependent on Vps45, a Sec1/Munc18 (SM) protein that participates in vesicle fusion in the *trans*-Golgi network-early endosome and the late endosome pathways via interactions with soluble N-ethylmaleimide-sensitive attachment protein receptor (SNARE) proteins [28]. A *vps45* mutant shows increased sensitivity to the ETC inhibitors SHAM (alternative oxidase), antimycin A (complex III), and potassium cyanide (complex IV). These results further highlight connections between endomembrane trafficking systems and mitochondria, building on earlier studies demonstrating, for example, that proteins for the ER–mitochondria encounter structure (ERMES) are required for virulence in *C*. *albicans* and *A*. *fumigatus* [30,31].

### Mitochondria participate in the response to stress conditions relevant to the host environment

Mitochondria also function in the response and adaptation of fungal pathogens to stress conditions in the mammalian host such as oxygen and nutrient limitation, elevated temperature, pH variability, and cationic, oxidative, and nitrosative stresses [3]. A fascinating example involves the “division of labor” observed for mitochondria of *Cryptococcus gattii*, a species complex closely related to *C*. *neoformans* [32]. In this case, a subpopulation of fungal cells adopts mitochondria with a tubular morphology in response to host reactive oxygen species (ROS), and these nonproliferating cells resist phagocytic killing and facilitate the intracellular proliferation of other cells with nontubular mitochondria. Recent studies have further defined conditions and factors that control mitochondrial fusion and morphology as well as stress resistance and virulence [33,34]. For example, mitochondrial fragmentation in *A*. *fumigatus* has been documented as a response to oxidative stress and as a measure of antifungal killing by human granulocytes [34]. Additionally, mutations that block mitochondrial fusion in *C*. *neoformans* cause loss of virulence in a mouse model and increased susceptibility to oxidative and nitrosative stress and ETC inhibitors [33].

Hypoxia is a relevant stressor due to the limited oxygen available to fungal pathogens in the host environment. The role of hypoxia has been well characterized for *A*. *fumigatus* and is emerging as an important aspect of *C*. *albicans* pathogenesis [35,36]. As with the response to low iron described above, hypoxia also provokes cell wall masking and evasion of the host immune response (i.e. polymorphonuclear neutrophil (PMN) killing and ROS); the cAMP/PKA signaling pathway is also required [21,36,37]. Mutations that impair mitochondrial function (e.g., loss of Goa1 or Upc2) decrease cell wall masking, while elevated ROS due to the loss of the alternative oxidase increases masking [36]. Connections between alternative oxidase and susceptibility to oxidative stress have also been documented for *A*. *fumigatus* and *C*. *neoformans* [35,38,39]. Interestingly, the lethality of *C*. *albicans* in a *Caenorhabditis elegans* worm model of infection was increased in hypoxic versus normoxic conditions [37].

Adaptation to elevated host temperature also involves mitochondrial functions. For example, loss of the temperature-responsive J domain protein Mrj1 impairs mitochondrial function in *C*. *neoformans*. Mrj1 supports ETC function, and the *mrj1* mutant displays a reduced ability to cause disease in mice, increased shedding of capsule polysaccharide, and cell wall defects [16]. The latter 2 phenotypes were recapitulated by treatment with antimycin A, a finding consistent with a positive role for Mrj1 in maintaining ETC function at the complex III step. In *C*. *albicans*, the J domain protein, Ydj1, participates in mitochondrial protein import and influences the yeast to hyphal transition, adaptation to elevated temperature, and sensitivity to osmotic, oxidative, and cell wall stress [40].

A number of regulatory factors influence mitochondrial functions and virulence in *C*. *neoformans*. For example, the transcription factor Mig1 regulates the expression of genes encoding mitochondrial functions, and a *mig1* mutant is sensitive to ETC inhibitors, oxidative stress, and agents that challenge cell wall integrity [41]. Similarly, Cheon and colleagues found that the transcription factor Gsb1 regulated genes for respiration and the cell cycle and that a *gsb1* mutant was sensitive to a variety of stresses (temperature, oxidative, nitrosative, osmotic, and cell wall) as well as ETC inhibitors [42]. This mutant was also attenuated for virulence in mice. More recently, mutations in the *CDK8* and *SSN801* genes encoding components of the kinase module of the mediator complex were shown to influence mitochondrial morphology, growth on acetate, and the response to oxidative and cell wall stresses [43]. In general, a pleiotropic impact of mitochondrial defects on the stress response and the ability to cause disease are common themes of these recent studies.

### Mitochondrial functions influence metabolic flexibility and adaptation to the host environment

Mitochondria in fungal pathogens are known to connect virulence to carbon source flexibility and the response to the host environment [44,45]. For example, the ability of *C*. *albicans* to grow on alternative carbon sources even in the presence of glucose provides the fungus with the ability to proliferate in different host niches and potentially compete with other microbes in the gut [45–47]. Defects in mitochondrial functions can reduce metabolic flexibility as illustrated by loss of Nuo2, a subunit of ETC complex I. The *nuo2* mutant is unable to grow on nonfermentable carbon sources and has reduced colonization of the gut, a phenotype that could be reversed by feeding mice a diet rich in glucose [48]. Signaling via the cAMP pathway to regulate the yeast to hyphal transition in *C*. *albicans* is also connected to mitochondrial metabolism of arginine, ornithine, and proline [49]. In this case, the metabolic response to the amino acids is linked to ATP levels and glucose availability. The connection between the formation of hyphae, cAMP/PKA signaling, ATP levels, and mitochondrial function has also been established with inhibitors of respiration and with mutants lacking components of the tricarboxylic acid cycle or the ETC [44,50,51]. Together, these studies highlight the importance of assimilating different carbon sources available in the host and responding to them as signals to elicit morphological changes required for colonization as a commensal or for pathogenesis.

### Mitochondria play a role in fungal dormancy

For fungal pathogens acquired by inhalation (e.g., *A*. *fumigatus*, *C*. *neoformans*, and dimorphic fungi), fungal cells can persist in the host in granulomas and phagocytic cells and can avoid the host immune response during the latent period [52]. Importantly, these fungal cells can reactivate and cause disease when the host immune system becomes impaired, e.g., in HIV/AIDS patients, after a latent period of months to years [52]. Mitochondria likely play a role in the latency or dormancy of fungal pathogens. For example, Alanio and colleagues identified a subpopulation of *C*. *neoformans* cells with features suggesting dormancy upon interaction with phagocytic cells in vitro and during murine cryptococcosis [53]. These features included slow growth in conventional media and altered expression of genes encoding mitochondrial functions. Further analysis by Hommel and colleagues identified conditions of nutrient limitation and hypoxia that generated viable but not culturable cells (VNBCs), and these cells could be reactivated for growth by pantothenic acid [54]. Mitochondria of the dormant cell population were transcriptionally active and displayed an increased mass and were mostly depolarized. Moreover, a multiomics analysis suggested a critical role of the fatty acid pathway in mitochondria in the dormant cells. These studies support the idea that mitochondrial functions contribute to adaptation to stressful conditions and the ability of fungal cells to enter a state of dormancy.

### Summary

It is clear that mitochondria are critically important for the ability of the major fungal pathogens of humans to cause disease. However, mitochondrial processes such as iron metabolism, respiration, and carbon metabolism are central to proliferation, making it difficult to evaluate specific contributions to virulence (Fig 1). This challenge is particularly well illustrated by the impact on the cell surface, the response to stresses encountered in the host, and aspects of morphogenesis. Additional studies are therefore needed to understand the integration of mitochondrial functions with virulence, to appreciate fungal-specific contributions of mitochondria, and to further explore the organelle as a potential therapeutic target.
